# Household

**Published:** 1889-05

**Authors:** 


### HOUSEHOLD.

Milk Soup.—Cut in large slices four potatoes and one onion. Place them in a sauce-pan over the fire with a piece of butter the size of a walnut, and allow them to steam for a few minutes; add half a pint of boiling water and simmer till the potatoes are soft. Mash them through a colander; add one pint of milk and return to the fire. Season with pepper and salt, and simmer, but do not boil. Serve with grated cheese. This quantity is sufficient for two or three persons.

De Crespigny Pudding.—Soak six ounces of bread crumbs in half a pint of milk; heat almost to the boiling point and add the beaten yolks of four eggs. Flavor with lemon, but add no sugar. Butter a pudding-dish and cover the bottom with a thick layer of jam of any kind. Pour in the other ingredients and bake half an hour in a slow oven. Beat the whites of the eggs to a stiff froth and stir in six tablespoonfuls of sifted sugar. Pile it up on top of the pudding and return to the oven for a few minutes. To be eaten with cream.

Dunnikier Orange Pudding.—Take five ounces of butter, melt it in a pan, but do not let it boil. Add to it while warm five ounces of sugar and the beaten yolks of ten eggs. Mix well together and beat thoroughly. Cover two pie plates with puff paste and spread over it a layer of orange marmalade. Pour over this the other ingredients, and bake about half a hour. Beat the whites of four eggs with six tablespoonfuls of sugar. Spread over the pudding and return to the oven till slightly colored.

Velvet Cake.—Beat the yolks of six eggs until frothy. Add two cups of granulated sugar, and beat for fifteen minutes. Add the well-beaten whites of three eggs, leaving the other three for the frosting. Stir into one cup of boiling water, two and one-half cups of flour, with one tablespoonful of baking powder mixed dry, flavor with lemon extract. Bake in three layers, and put this icing between them. The whites of three eggs, well eaten, six dessert spoonfuls of pulverized sugar to each egg. Beat stiff, and flavor with lemon.

Rice Croquettes.—Boil the rice until quite soft and tender; while warm, measure; to every teaspoonful of boiled rice add an egg, well beaten, a tablespoonful of butter, pepper and salt to taste, and a half-teacup of any kind of cold fresh meat, ham or tongue, chopped fine. When cold, with floured hands make into croquettes, cover with beaten egg, roll in cracker dust, and fry in hot drippings until nicely browned.

Baked Fish —Let the fish remain in cold water, slightly salted, for an hour before time to cook it; place the gridiron on a dripping pan with a little hot water in it and bake in hot oven ; just before done butter it well on top, and brown nicely. The time of baking depends upon the size of fish. A small fish will bake in about half an hour, and a large one in an hour. They are very nice when cooked as above and served with a sauce which is made from the gravy in the dripping pan, to which is added a tablespoon of catsup, and another of some pungent sauce and the juice of a lemon. Thicken with brown flour moistened with a little cold water. Garnish handsomely with sprigs of parsley and currant jelly.

